# Capitalist Discourse, Subjectivity and Lacanian Psychoanalysis

**DOI:** 10.3389/fpsyg.2016.01948

**Published:** 2016-12-09

**Authors:** Stijn Vanheule

**Affiliations:** Department of Psychoanalysis and Clinical Consulting, Ghent UniversityGhent, Belgium

**Keywords:** psychoanalysis, Lacan, discourse, capitalism, ADHD, autism, neurosis, psychosis

## Abstract

This paper studies how *subjectivity* in capitalist culture can be characterized. Building on Lacan's later seminars XVI, XVII, XVIII, and XIX, the author first outlines Lacan's general discourse theory, which includes four characteristic discourses: the discourse of the master, the discourse of the university, the discourse of the hysteric and the discourse of the analyst. Next, the author explores the subjectivity and the mode of dealing with jouissance and semblance, which is entailed in a fifth type of discourse, the capitalist discourse, discussed by Lacan (1972). Indeed, like the other discourses that Lacan discerns, the discourse of the capitalist can be thought of as a mode of dealing with the sexual non-rapport. It is argued that in the case of neurosis the discourse of the capitalist functions as an attempt to ignore the sexual non-rapport and the dimension of the unconscious. Psychosis, by contrast, is marked by an a priori exclusion from discourse. In that case, consumerist ways of relating to the other might offer a semblance, and thus the possibility of inventing a mode of relating to the other. Two clinical vignettes are presented to illustrate this perspective: one concerning the neurotic structure and one concerning the psychotic structure.

## Introduction

The idea that a consumerist culture has permeated the fabric of society is nothing new. Not only is there little public discussion on alternatives for the free market, this model has pervaded our social-cultural life (McGowan, 2004; Sennett, 2006; Verhaeghe, 2014; Mura, 2015). Several authors suggest that capitalism imposes the customs of market-oriented thinking on all domains of our life, profoundly affecting the way we experience and make sense of ourselves and others. Indeed, nowadays capitalism shapes our experience of subjectivity. Marcuse (1964) suggests that capitalism integrates people in a model of consumerism, where they work and consume more than they need. This not only has damaging effects on the environment and on social life, but also contaminates mental life by installing false needs that people want to satisfy. Sennett (2006) suggests that contemporary capitalist culture implies a passion for self-destruction linked to increased consumption and Verhaeghe (2014) argues that, with their strong focus on success and happiness, both capitalism and neoliberal culture destabilize the experience of identity.

In this paper I focus on the later work of Jacques Lacan to study how we can characterize the relation between the subject and the other, and between subject and object in a capitalist culture. In the late 1960s and 1970s, Lacan occasionally discussed the impact of capitalist culture on subject formation. In line with his general idea that the human subject comes into existence through the play of signifiers^1^, which originate from the symbolic order, in this period of his work he also assumed that the *symbolic order* of capitalism molds the subject in a particular way. Capitalist culture affects the way we deal with distress and suffering; it shapes the way we relate to others; it determines the way the unconscious functions; and it influences the kind of request for help that an individual might extend to a psychoanalyst. Indeed, early in the nineteen seventies Lacan (1972) indicated that the capitalist discourse had started to replace the traditional discourse of the master. The classic figure of the other, which largely rests on the structure of the discourse of the master, had faded away (Žižek, 1999), affecting the subject to the extent that a reconsideration of how we work clinically is needed (Miller, 1993).

As I take up Lacan's line of reasoning, my interest is mainly clinical. Other authors have studied Lacan's discussion of capitalism in terms of his overall engagement with the work of Karl Marx (e.g., McGowan, 2004; Bryant, 2008; Pavón-Cuéllar, 2009, 2011; Bruno, 2010; Tomšič, 2015). My objective differs in that I focus on the structure of capitalism as articulated in Lacan's statements on the discourse of the capitalist (*le discours du capitaliste*), and on its clinical implications. In 1972 Lacan considered this discourse to be an amended version of the discourse of the master.

Building on Seminars XVI, XVII, XVIII, and XIX, I first outline Lacan's overall discourse theory. I explain the four positions of each discourse, the initial four discourses that he discerns (i.e., discourse of the master, the university, the hysteric and the analyst), and further changes that Lacan made to his theory as he focuses on semblance, jouissance, surplus-jouissance, and non-rapport. Following this, I explore *how the subject takes shape* in the discourse of the capitalist and the *mode of dealing with jouissance* that Lacan's fifth discourse entails. Indeed, just like the other discourses that Lacan discerns, the discourse of the capitalist can be thought of as a mode of dealing with the *sexual non-rapport*. Finally, the clinical implications of working with this discourse are presented. On the one hand I review Lacan's reflections on the figure of the saint, which he contrasts with the capitalist, and proposes as model for the psychoanalyst. On the other hand I discuss two clinical vignettes; one that concerns the neurotic structure and one that concerns the psychotic structure. In both cases I discuss how the capitalist discourse takes shape, and point out that whereas in the context of neurosis, the capitalist discourse bears witness to a strategy of ignorance, it might have a stabilizing effect in psychosis.

## Elements of Lacanian discourse theory

**Figure 1:**
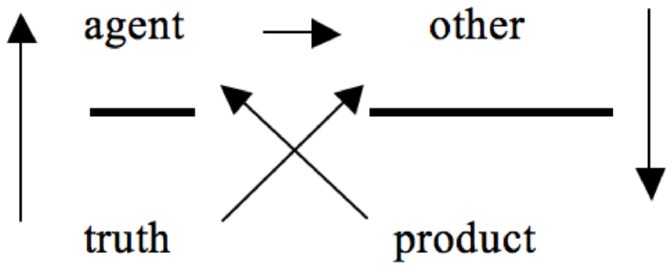
**The overall structure of the discourses and the relations between all positions (based on Lacan, 1969–1970, p. 93, 196; Lacan, 1972, p. 40)**.

Lacan (1969–1970) first elaborated his discourse theory in Seminar XVII, where he discerns four discourses^2^: the discourse of the master, the discourse of the university, the discourse of the hysteric and the discourse of the analyst. These four discourses all have the same structure, and consist of four positions: *agent*, top left; *other*, top right; *truth*, bottom left; *product*, bottom right (Figure 1)^3^ connected via a fixed set of arrows.

What is essential to the four discourses is that a desiring^4^ “agent” addresses an “other,” which is indicated by the horizontal upper arrow. In the move from “agent” to “other” we recognize the human tendency to create social bonds. However, here Lacan is not expressing some sort of romantic view on human interrelations, but is stressing that the relationship between “agent” and “other” is marked by a “disjunction of impossibility” (Verhaeghe, 2004, p. 59; Bruno, 2010): the message that the agent sends is never received as it was intended. Lacan (1969–1970, p. 174) explains this as follows: “The first line comprises a relation, indicated here by an arrow, which is always defined as impossible. In the master's discourse, for instance, it is effectively impossible that there be a master who makes the entire world function. Getting people to work is even more tiring, if one really has to do it, than working oneself.” Indeed, the agent's address never provokes a reciprocal reaction, which is why no returning arrow connects the “other” back to the “agent” (see Figure 1).

The lower part of the formula highlights the hidden side of discourse. The first position on the bottom left is “truth,” which is connected to the position of the “agent” by an arrow pointing upwards. This arrow indicates that all actions made by the agent in a given discourse rest on a hidden truth. Indeed, characteristic of all discourse is that a repressed element motivates the agent's actions, and that this repression engenders the possibility of a social bond, represented at the upper level of the discourses. In a similar vein, “truth” also has an effect on the position of the “other,” which Lacan emphasizes by drawing an additional (diagonal) arrow.

The arrow pointing downwards (right side of Figure 1) indicates that the agent's address to the other has effects: a “product” is created. This product fuels the agent, but occupies a disjunctive position in relation to the truth that set the discourse in motion.

**Figure 2:**
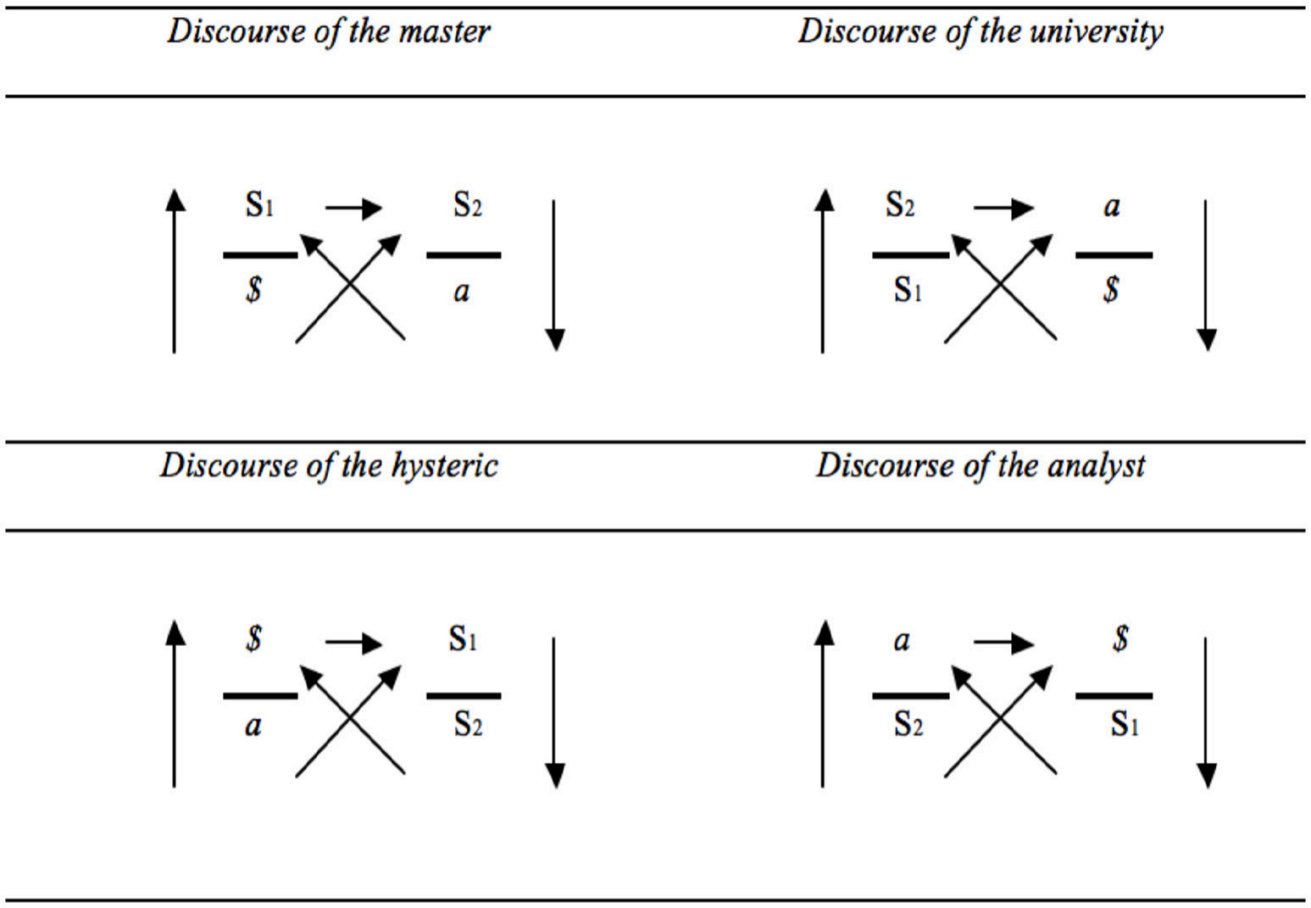
**Discourse of the master, discourse of the hysteric, discourse of the university and discourse of the analyst (based on: Lacan, 1972, p. 40)**.

In the discourse of the master (see Figure 2), a master signifier (S1) is formulated by the agent, and imposed onto the other who is presumed to function by means of knowledge (S2). Characteristically, such a domineering move rests on the repression of subjective division (*$*), and as a product the other is reduced to the position of an object (*a*). For example a therapist may tell his phobic client to be brave (S1) and to face the crowds he is afraid of by adhering to specific instructions as to how one might behave in groups (S2). By adopting such a directive style the therapist puts his own uncertainty in social situations (*$*) aside, and by obeying the therapist, the client is reduced to a pawn in the game of social interactions, which will finally produce further discontent (*a*) that might engender the formulation of new directives (S1).

Central to the discourse of the hysteric is the active formulation of complaints (*$*) and the search for an other who is presumed to have an answer (S1) for what bothers the subject. This discourse represses the truth that all desire rests on a lack that cannot be alleviated (*a*), and typically results in the production of narratives (S2) that don't solve the fundamental lack (*a*), but actually engender further irritation (*$*).

The discourse of the university builds on the proclamation of knowledge (S2). Such knowledge always rests on the acceptance of dogmas and assumptions (S1), but this is neglected in this discourse. Characteristically, the other is put in the place of the object (*a*). This produces discontent (*$*), which fuels further knowledge creation (S2).

Finally, in the discourse of the analyst, the analyst qua agent confronts the other with a so-called object *a*, logically notated *a*. The object *a* refers to a drive or jouissance-related remainder that cannot be named and that fuels desire^5^. For example, the analyst's silence, which often baffles the analysand who expects reciprocity in the interaction, can function as an object *a* (see Lacan, 1971, p. 25). By occupying the place of the object *(a)* the analyst creates a place where, via free associative speech, subjective division can be articulated (*$*). In order to pay close attention to the singularity of the patient the analyst puts aside pre-established ideas about patients and pathologies (S2), such that key signifiers that mark the analysand's subjectivity (S1) can be formulated, which fuels the analyst's positioning qua object *a*.

## Semblance and jouissance in discourse

**Figure 3:**
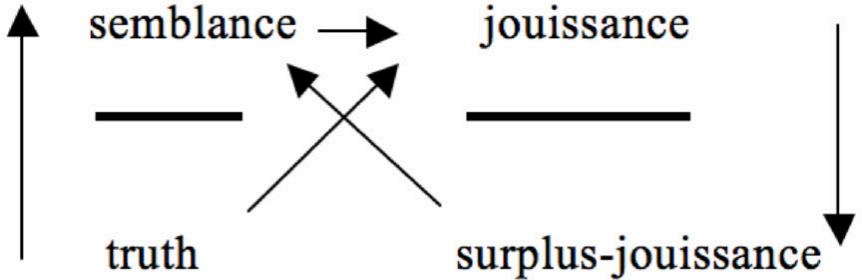
**Overall structure of the discourses Lacan discerns in Seminar XIX (based on Lacan, 1971–1972, p. 67, 193; Lacan, 1972, p. 40)**.

In Seminar XVIII (e.g., Lacan, 1971, p. 25) and Seminar XIX, Lacan (e.g., Lacan, 1971–1972, p. 67) somewhat rearticulated the positions he first entitled as “agent,” “other,” and “product” (see Figure 3), indicating that engaging in discourse above all means that one makes use of semblance. During his teaching Lacan interpreted the concept of semblance in various ways (Grigg, 2007). In the nineteen fifties he uses the concept *semblance* (“*le semblant*”) to refer to the world of appearances that is installed by means of the Imaginary. At that moment semblance is an imaginary phenomenon that needs to be distinguished from the Symbolic. As Lacan developed his discourse theory this all changed profoundly. At this point he suggests that the fact of social relations as such implies semblance, which is expressed in the following statement: “discourse as such is always discourse of semblance” (Lacan, 1971–1972, p. 226, my translation). Henceforth, discourse unfolds when someone forges a position in relation to another; semblance is “the proper object based on which the economy of the discourse regulates itself” (Lacan, 1971, p. 18, my translation). For example, the discourse of the master takes shape if someone plays the role of the commanding agent.

In seminar XIX, the position of the other is described as the position of *jouissance* (Lacan, 1971–1972, p. 193). Here Lacan defines jouissance as a disturbing dimension in the experience of the body, which renders the subject unable to experience itself as a self-sufficient enjoying entity (Lacan, 1971–1972, p. 217). Jouissance is immensely disruptive. It is a dimension of otherness that we all have to deal with. Indeed, the very idea of “dealing with it” bears witness to discourse; that is, to the fact that we treat jouissance by making an appeal to an agent or semblance, which is expected to manage it: jouissance provokes the mobilization of semblance. The root of jouissance is in the structurally dysfunctional status that the body has for the human being (Lacan, 1971–1972, p. 217).

What is typical for discourse, is that it envelopes a semblance around jouissance, and as a result jouissance is no longer unlimited, but conditioned by the element occupying the position of semblance. In this maneuver, a social bond is created: “What is discourse? It is that which, in the arrangement of what might be produced because of the existence of language, makes up the function of the social bond” (Lacan, 1972, p. 51, my translation).

## Surplus-jouissance as the product of discourse

In the early nineteen seventies Lacan frequently points out that the product of discourse makes up a “surplus-jouissance” (e.g., Lacan, 1971–1972, p. 193). In forging his concept “surplus-jouissance” Lacan builds on Marx's concept “surplus value.” In Marx's *Capital* (1999), the notion of surplus value is defined as the difference between the exchange value of products of labor (commodities) and the value that coincides with the effort of producing these products, i.e., the means of production and labor power. In our market economy system, Marx says, money is the pre-eminent criterion to measure the amount of the value that is realized. Within the capitalist system gaining surplus value seems to be the sole aim. Profit-making and the expansion of capital are the motives that drive capitalism. However, gaining surplus value is only possible by selling fetishized commodities for a price that is higher than the value attributed to labor that produced them. If equivalent values are exchanged, no surplus value can be realized.

Marx indicates that the realization of this aim depends on a trick, and it is this cunning trick that interests Lacan (1968–1969, pp. 64–65; Vanheule and Verhaeghe, 2004). In the market the capitalist buys labor power in order to produce merchandise. Marx states that the trick put into practice in this process is that the capitalist pays the laborer as much as he has to, but less than the market value of what the laborer actually produced. In other words, in the process of exchanging value (labor power/money) the capitalist pockets a monetary surplus behind the back of the laborer, and behaves as if he too worked hard during the process of production. Here Marx states that the capitalist must hide his smile: “after a hearty laugh, he re-assumes his usual mien” (Marx, 1999, p. 126). This laughter results from the fact that the value that is created during a workday is actually much higher than what the capitalist pays the laborer.

Capitalist production implies that one no longer works solely in order to satisfy needs, and stops once they have been met. Production continues beyond satisfying needs, which results in a fetishist relation to surplus value (Tomšič, 2012, 2015). Lacan (1968–1969, pp. 64–65) concludes that the secret gain of surplus value is both the product and the motor of the capitalist production system. Yet, despite the appropriation of surplus value, Marx stresses that the capitalist does not personally enjoy what he gains. The capitalist is only the support that makes the system run. Therefore, what the capitalist system produces are suppositions and phantasies of gratification, while in fact nobody enjoys (McGowan, 2004). Indeed, this is what Lacan also stresses when addressing Marx's socio-economic analyses: “There is only one social symptom: each individual actually is a proletarian” (Lacan, 1974, p. 187, my translation).

Furthermore, Lacan suggests that the general structure of discourse is “homologous” to the system of capitalism described by Marx, and this is why the above discussion of surplus value is relevant. Both systems produce an element of excess, in relation to which a fetishist relation is created. In capitalist production surplus value and/or commodities are fetishized, while in the use of discourse a fetishist relation with surplus-jouissance (*plus-de-jouir*) is created (Lacan, 1968–1969, p. 45; Tomšič, 2012).

Homology means that their structure is identical (Regnault, 2005): while coming in a different form, the use of discourse and capitalist production obey the same logic. As we use discourse language is produced, in the capitalist system commodities are produced. Yet, through the process of exchange something is lost. By using discourse one is robbed of something: in attempting to address jouissance by means of language, and find a solution for it through the social bond, the experience of an un-articulated “beyond” is produced. Using signifiers to name jouissance confronts the speaker with *a dose of corporeal tension* that is not inherent to language: a surplus-jouissance that can only be located in phantasy or delusion comes to the fore. It is precisely at this point that the function of laughter can be situated. In Marx's system, laughter refers to the capitalist's gain of surplus value, and to the process of alienation that this entails. In the use of discourse, laughter refers to the surplus-jouissance inherent in our alienation in the signifier.

In explaining surplus-jouissance, Lacan points to the joke. As we speak we invariably also utter nonsense, and because of this we laugh. Yet, why exactly does the joke provoke laughter? Lacan (1968–1969, p. 64, my translation) suggests the following: “it [the joke] provokes laughter, in the end, to the extent that it is actually hooked to the failure inherent to knowledge.” The pursuit of meaning through speech implies deadlocks. Speech is always a half-saying (*mi-dire*). It misses its point, and this failure coincides with a dose of jouissance, to which laughter bears witness. Consequently, surplus-jouissance has a status of lack and loss (Tomšič, 2015)—language use always misses the point; expressed by downward arrow in the formulae for discourse—and at the same time makes discourse function as an endless attempt to get hold of what one misses; expressed by the diagonal arrow from surplus-jouissance/product to semblance/agent. Furthermore, by connecting the manifestation of surplus-jouissance to laughter and misrepresentation, Lacan situates surplus-jouissance at the level of the unconscious (Lacan, 1971, p. 21). In Marx's production system the capitalist laughs with the money the system generates; in Lacan's model the user of discourse laughs to the extent that, at the level of the unconscious, a surplus of jouissance is produced which one fails to get hold of. The unconscious concerns the combined expression of half-saying and surplus-jouissance.

In the discourse of the master the object *a* is a component of libidinous corporeality that is delineated by the use of signifiers, but is not represented by means of the signifier. It is what remains leftover after imposing knowledge (S2) onto jouissance. Qua element of symbolic nothingness, the object *a* nonetheless makes itself felt as corporeal tension, gravitating around a gaze, a voice, or in the element of oral nothingness to be taken in, and anal nothingness to be given away. In the end, this surplus-jouissance is juxtaposed with the master signifier (S1), but, as mentioned previously, it doesn't correspond to the truth that the discourse was initially fueled by. In the end the discourse of the master stresses the fact that there is no hope that subjective division can ever be transcended, or that discontent can be resolved if we address jouissance by means of language, which is what we typically do. *Unbehagen* is structurally unsolvable, which is expressed in the formula by the fact that none of the arrows arrive at *$*. It is precisely the failure that coincides with the discourse of the master that, in Lacan's reasoning, makes analytical discourse possible. Through the exploration of subjective discord via free association, there is a return in the analysis to the signifiers that connote and mark the subject.

In most discussions of surplus-jouissance, Lacan starts from the master discourse. In the discourse of the master the object *a* is the surplus that the semblant is confronted with. Yet, in terms of Lacan's later discussions of the structure of discourse (Figure 3), surplus-jouissance is not identical to the object *a*, but the end position of each discourse (Lacan, 1971–1972, p. 193). In the discourse of the university the divided subject occupies this place; in the discourse of the hysteric it is unconscious knowledge that emerges; and in the discourse of the analyst the master signifier makes up the surplus-jouissance.

## Non-rapport is the missing fundament of all discourse

**Figure 4:**
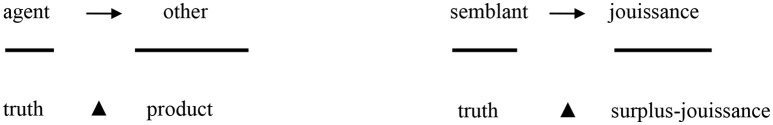
**Overall structure of the discourses that Lacan discerns, including the non-rapport at the basis of the relationship between agent/semblant and other/jouissance (based on: Lacan, 1969–1970, p. 151)**.

The outcome that discourse creates is always at odds with the truth that first mobilized the turn to the Other. This is what the absence of an arrow between “surplus-jouissance”/“product” and “truth” indicates (Figures 1, 3): the consequence of discourse has nothing to do with the truth that first set it in motion. Conversely, the product of discourse does not leave the agent or semblance unaffected, which is what the diagonal arrow pointing from “product” back up to the “agent” refers to. Indeed, Lacan repeatedly stresses that the invariant experience of impossibility that characterizes the connection between agent/semblance and other/jouissance builds on a fundamental *non-rapport*. Underlying the relation between agent/semblant and other/jouissance a fundamental non-rapport can be found between truth and product/surplus-jouissance, which Lacan sometimes marked by a small triangle (▴)(Figure 4) or a double slash (//).

In this line of reasoning the concept “rapport” has a specific meaning. Lacan defines the relationship between two elements in terms of a “rapport” if the laws that govern their bond are fixed. If this is the case, the relationship “can be written” (Lacan, 1971, p. 65, my translation). In this view, gravitation is a relationship that can be written: starting from knowledge of the physical properties of an object, like its mass and its density, formulas make it possible to calculate how long it will take to touch the ground when the object falls from a given height.

Applied to sexuality it could be argued that the way male and female animals interact is fairly uniform, and depends only marginally on how two specific specimens behave. Yet as soon as our focus is on humans, the nature of relationships is not a priori given. In this context Lacan (1971–1972, p. 18, my translation) notes “the inability to formulate a precise rule at this point.” A sexual relation is not installed on the basis of the correct triggers being projected, but is always contingent, and shaped through the encounter between two speaking beings: “So it is in a discourse that natural men and women, as one might say, have to valorize themselves as such” (Lacan, 1971, p. 146, my translation). Thus, in humans the sexual relationship cannot be written: it cannot be formalized in terms of fixed rules that apply to each particular relationship: “The Other is absent from the moment that what is at stake is the sexual relationship” (Lacan, 1971–1972, p. 104, my translation). That's why Lacan qualifies the sexual relationship as a non-rapport. In this context he also mentions a “deficiency of the sexual rapport” (Lacan, 1971, p. 167, my translation), which indicates that there is no signifier that might name what a sexual relation consists of. The only things humans are left with are speech and discourse, which for Lacan (1971, pp. 83, 148) should be thought of as an effect of the non-rapport. Indeed, all speech on the sexual non-rapport is a mere “half-saying” (Lacan, 1971–1972, p. 12, my translation), meaning speech that is ever besides the point. The fundamental inability that is inherent to the sexual relationship cannot be solved by means of the signifier: “sexual encounters always fail” (Lacan, 1971–1972, p. 27, my translation), which produces a state of desperation (Lacan, 1971–1972, p. 115). The only option individuals are left with is inventing ways of dealing with the non-rapport.

As a consequence, in Lacan's view the sexual relationship is Real. It is “that which does not stop not being written” (Lacan, 1972–1973, p. 57, my translation). This implies that in order to establish a bond between individuals, speech must always be mobilized. Indeed, given the non-rapport at the basis of all relationships, we cannot but make use of discourse, which opens up a field of semblance: “For the boy, what is at stake in adult age, is acting-a-man [*faire-homme*]. This is what constitutes the relation to the other party…one of the essential correlates of this acting-a-man, is to indicate to the girl that one is so. In a word, we find ourselves put right away in the dimension of the semblance” (Lacan, 1971, p. 32, my translation). A male person is not automatically “man” in relation to a woman. Only by manifesting oneself as a man or a woman in discourse can a bond between partners take shape. In other words: *the* sexual relationship is Real and cannot be written; what remains open is the possibility of engaging oneself in *a* sexual relation. This is only possible through the use of discourse (see also Vanheule, 2014).

## Capitalist discourse^6^

From the late 1960s on, Lacan occasionally commented on the particularities of capitalist discourse, highlighting how it differs from the four discourses he first discerned. A systematic discussion or theory on this discourse cannot be found in his work. In this section I provide a reading of Lacan's work on this topic, and aim at formulating a more comprehensive idea on the particularities of the capitalist discourse. Other interpretations of this fifth discourse can be found in the works of Bruno (2010), Bryant (2008), Pavón-Cuéllar (2009), and Tomšič (2015). As Bianchi (2012) correctly indicates, with his fifth discourse Lacan gives a non-Marxian account of capitalist culture, stressing the logic of consumption rather than the mode of production that capitalism implies. This is also how I interpret Lacan's capitalist discourse. Later interpretations of Lacan's fifth discourse do not focus only on the dynamics of consumerism, but make explicit links with Marxism and explore dynamics of production as well (e.g., Bryant, 2008; Pavón-Cuéllar, 2009).

**Figure 5:**
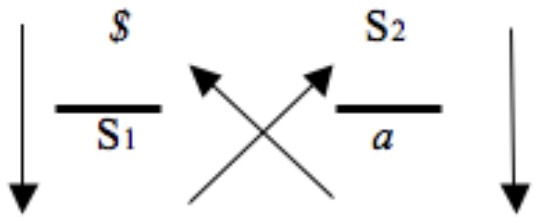
**Capitalist discourse (based on: Lacan, 1972, p. 40)**.

On one occasion, during a 1972 lecture at the University of Milan, entitled *du discours psychanalytique*, Lacan articulated a model on the precise structure of capitalist discourse (see Figure 5). This model coheres with Lacan's initial four discourses, but cannot be seen as just another variant in the series of discourses. After all, Lacan's four discourses have a strict structure: four positions are linked by means of five unidirectional arrows (Figures 1, 3); and 4 elements (*$*, S1, S2, and *a*) rotate in a fixed order across these positions (Figure 2). The discourse of the capitalist disrupts this structure, and is a “mutant” of the discourse of the master. Indeed, Lacan (1972, p. 48) understands capitalist discourse as the contemporary variant of the classic discourse of the master. Yet with regard to the discourse of the master, it contains 3 mutations^7^ (Lacan, 1972, p. 40):

1. *$* and S1 exchange places.
2. The arrow pointing upward on the left that makes the position of the truth unattainable in the classic discourse changes now into an arrow pointing downwards.
3. The upper horizontal arrow that made the connection between “agent” and “other,” or “semblance” and “jouissance,” disappears.

The effect of these three changes is that a number of obstructions that are inherent to the four discourses are not characteristic of the fifth discourse. We can circulate within the capitalist discourse like go-carts on a racetrack. Indeed, in the capitalist discourse, the non-rapport is circumvented. Tomšič (2015, p. 220) describes this as follows: “The vectors show that the capitalist discourse is grounded on the foreclosure of the impossibility of totalization that marks other discourses, an impossibility that is structurally determined by the fact that the signifiers constitute an open system of differences.”

Specifically, in the four standard discourses the position of truth is not targeted by an arrow, and the positions of “agent”/“semblance” and “other”/“jouissance” are influenced by two (not mutually related) other positions, which makes its functioning structurally lapse. In the capitalist discourse, “a very small inversion between the S1 and the *$*, which is the subject, is enough for it to run as if it were on wheels, it can't run better, but it actually runs too fast, it runs out, it runs out such that it burns itself out”^8^ (Lacan, 1972, p. 48, my translation). Indeed, what is structurally characteristic of the discourse of the capitalist is that while the four positions remain intact, the pathways made up by the arrows change: in all positions one arrow arrives, such that a closed circuit of arrows is created. The structural lapse that marks the four standard discourses cannot be found at the root of this fifth discourse, which, so to speak, makes it run on wheels. Yet Lacan suggests that in the end the one functioning along the lines of this smoothly running process burns himself out, and gets consumed. One idea that the above quote articulates, is that in the capitalist discourse subjectivity is corrupted. The main structural reason for this is that in this discourse, the distance between *$* and *a* is lost: *corporeal tension* that is proper to surplus jouissance *disturbs the subject*.

Just like in the discourse of the hysteric *$* is situated at the level of the agent/semblance. Indeed, the discourse of the capitalist essentially starts from the experience of subjective division. In line with his earlier work, Lacan suggests that the subject is, on the one hand, a connotative effect of language use—“the signifier is what represents a subject for another signifier” (Lacan, 1972, p. 51, my translation). On the other hand, the subject is determined by the object *a*, which is the structural cause of desire—“The object *a* is the true support of what we have seen function, and it functions so in a more and more pure way to specify each in his desire” (Lacan, 1972, p. 52, my translation). Yet, most characteristically, man is marked by sexuality, which is not instinctively organized, and makes up “that in which man never feels at ease at all” (Lacan, 1972, p. 38, my translation). In the discourse of the hysteric the *Unbehagen* thus obtained results in an address to the other. Capitalist discourse, by contrast, does not capitalize on the social relation: “capitalism, that was its starting point: getting rid of sex” (Lacan, 1974, p. 34). Indeed, the capitalist discourse directly aims at the root of the problem, which is what the downward arrow on the left indicates. This discourse does not encapsulate the discomfort of subjective division as structural, but aims to recuperate discontent in its very system. It is a discourse in which there are answers for this discomfort: there exists an S1 that answers the *$* and functions as a truth for the divided subject. For example, in our contemporary Western consumption culture, discontent is often deemed the upshot of having not yet obtained the right object and suggests that a state of subjective satisfaction will be reached once this object is obtained. In other words, the semblance of being dissatisfied can be answered with the S1 of a brand name or a product that offers the promise of satisfaction. Capitalist discourse actively cultivates the semblance of dissatisfaction, as well as a fantasy of self-sufficiency, completeness and vitality (Tomšič, 2015). The market^9^ tells us what we need: the merchandise it provides. These are all S1's: they are isolated signifiers that consumers take to be the truth of their discontent. Indeed, within the capitalist discourse, the products that make up the market constitute a despotic truth to which the subject is subjected.

The move from *$* to S1 reflects a denial of the structural quality of subjective division. On the one hand the capitalist discourse starts from subjective division, yet, on the other hand the move toward S1 suggests that subjective division might be overcome through alienation in a master signifier. This bears witness to a perversion-like movement: while in perversion “the subject takes care himself to compensate for the flaw of the Other” (Lacan, 1968–1969, p. 265, my translation), in capitalist discourse an S1 is carefully promoted to compensate for the flaw of the subject. In both cases, subjective flaw is believed to be corrigible, which is why the discourse of the capitalist is often described in terms of a generalized perversion (Mura, 2015). In line with this interpretation Lacan (unpublished document, 6 January 1972 meeting, my translation), postulates a rejection of symbolic castration at the basis of the discourse of capitalism: “What distinguishes the capitalistic discourse is this: Verwerfung, rejection, rejection outside all fields of the symbolic …of castration.” Within the capitalistic logic, the lack at the heart of subjectivity is not seen as a structural consequence of using signifiers, but an accidental frustration that can be remedied within the market of supply and demand. The assumption that an S1 exists for each discomfort is ingrained in this discourse.

As a result, capitalist discourse implies a *particularization of desire*, treated as if it is a demand. Whereas in classic discourse desire is singular in that it cannot be solved by means of the signifier, the capitalist discourse suggests that particular solutions for dealing with subjective division actually exist: the market is there to satisfy customers' demands. Consequently, at the point of desire, the capitalistic logic leads to exploitation: “the exploitation of desire, this is the big invention of capitalist discourse” (Lacan, 1973a, p. 97, my translation). This discourse exploits desire by treating it as a specific question to be answered by means of practical solutions. The superego command characteristic of capitalist times concerns an obligation to satisfy desire via consumption (McGowan, 2004). The market provides streams of products and services that are there to answer peoples' demands. Lacan suggests that this has a tranquilizing effect: “we couldn't do anything better so that people comport themselves with a little tranquility” (Lacan, 1973a, p. 97, my translation). However, as we will see later, this also happens at the point of subjectivity for a certain price.

Interestingly, following Žižek, Bryant (2008, p. 13) suggests that under the regime of capitalism, the subject's principal question is not “what do I desire” but “what *should* I desire,” which is “not a question about objects, but a question of those conditions under which the subject might be desired by the Other.” Indeed, it is a basic Lacanian tenet that the desire governing the subject is essentially mediated by the desire of the Other: “man's desire is the Other's desire” (Lacan, 1960, p. 690). Within capitalist discourse this implies that merchandise will not so much be preferred for its intrinsic qualities, but in terms of how it is evaluated by the other. Indeed, this is often how marketing proceeds, products are presented as highly desired by celebrities, which directs the consumer's desire.

Obviously, such exploitation of desire only works because the S1 that the capitalist discourse formulates as an answer is not at all random: S1 refers to an entire knowledge apparatus, S2, which guarantees the adequacy of the answer. Indeed, according to Lacan (1969–1970), there is compatibility between contemporary science and the capitalist discourse. In his view, the capitalist's discourse is engaged in a “curious copulation with science” (Lacan, 1969–1970, p. 110). Science ensures^10^ the development of S2, through which S1 grows ever more innovative and, as a result, old answers must be constantly replaced by new ones. Within the capitalist discourse, S1 is not a fixed anchorage, but a solution that is replaced by endlessly better solutions. The fact of the matter is that the innovation of S2 continuously recreates both S1 and the demand. The only thing that the system needs is the consumer: subjects that are prepared to translate their discord *$* in terms of the gap in the market that is delineated by S2, and who believe in S1.

**Figure 6:**
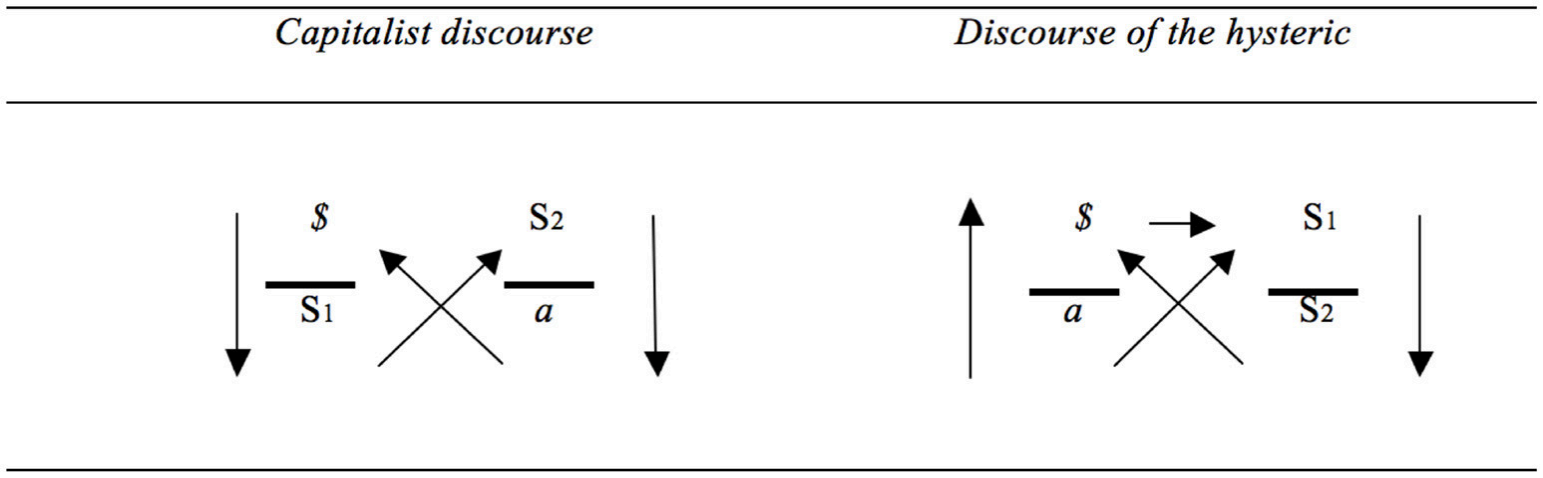
**Capitalist discourse and discourse of the hysteric (based on: Lacan, 1972, p. 40)**.

Moreover, the switch between S1 and *$* reveals something about what is taken seriously. In the discourse of the master, it is a signifier that is taken seriously: an S1 is adopted, and around this signifier a world of semblance is created through which the other and jouissance are addressed, which is what the upper horizontal arrow indicates. In the discourse of the capitalist, by contrast, it is discontent that is taken seriously. In this respect, the capitalist discourse resembles the discourse of the hysteric (Figure 6).

However, in the discourse of the hysteric, discontent is addressed to another who, as an authority, is supposed to have the answer (S1). This is expressed by the horizontal arrow. Such a move in the direction of the other remains absent in the discourse of the capitalist. In the discourse of the hysteric the move toward the other socializes or communalizes discontent. Discontent becomes an issue for which the other is equally responsible. Along this way, desire qua irresolvable dimension that needs to be recognized by the other is created. A classic example of this can be found in the butcher's wife's dream discussed by Freud in The Interpretation of Dreams. The woman in question found nothing more pleasurable than eating caviar, but forbade her husband to give it to her. In the same way, this woman knows that her friend likes nothing better than salmon and has a dream in which she cannot buy salmon. In this sense, refusing the object of satisfaction protects her subjectivity. It allows her to articulate questions over and beyond the fish and its eggs: the question as to what makes a woman attractive and the question of what her husband desires. Questions of this type can only be articulated to the extent that instant satisfaction is rejected. The preservation of the lack creates space for questions about love and identity.

The capitalist discourse follows a different logic, in that it takes desire as if it was a frustrated demand. It translates desire into solutions, meaning desire is not seen as the support of subjectivity, but merely seen as a demand that should be gratified. Why all the misery, the capitalistic logic would ask: caviar or salmon can simply be bought, right? In this way, what the discourse of the capitalist fills is the space for questions pertaining to love and desire: Lacan states “Any order, any discourse that resembles capitalism leaves aside what we will simply call the things of love, my friends. You see, that's just nothing” (Lacan, 1971–1972, 6 January 1972 lecture, my translation). Within this discourse subjective division is not seen as a manifestation of subjective truth along the lines of the unconscious, but as a state of permanent crisis the subject needs to get rid of (Pavón-Cuéllar, 2009).

When everything is gratification of demands, something at the root of the social bond gets lost (Declercq, 2006). The search associated with living with questions of existence (“who am I?” “what do you want from me?”) is replaced by a search for solutions in dealing with corporeal tension, and for experiences of fulfillment. Yet in Lacan's view, the net result that the discourse of the capitalist and master end up with is similar. Both end up producing the object *a*; a component of nonsensical libidinous corporeality that is created by using signifiers, but not represented by means of the signifier.

How can we understand this idea of producing an object *a* by means of capitalist discourse? Characteristically, this discourse implies a consumer (*$*) purchasing commodities or services at the market (S1), paying for them according to the *exchange value* the market dictates. Yet, once the consumer possesses or consumes the product, it is reduced to its *use value*. The S1 the consumer possesses is now brought into dialogue with the other signifiers that populate his world (S2). This does not so much create pleasure or fulfillment, but always implies a process of sobering up: in the end the consumption product is just an artifact among artifacts; a signifier among signifiers. Something that was of value when purchasing the product, which was ascribed to the product when it was still on the market, and which is no longer there when the consumer possesses the product: the commodity never delivers the hoped for satisfaction and “is always surrounded by a halo of disappointment in which unrequited desire painfully persists” (Bryant, 2008, p. 14). The anticipated glamor is lost, which is why the product has only one destiny: waste. Therefore, in line with Mura (2015, p. 166), it could be argued that “the production and consumption of success and satisfaction are consubstantial in the discourse of the capitalist with the production and consumption of both failure and emptiness.” What is produced is surplus-jouissance, which, in the next step, fires the *Unbehagen* or crisis at the level of the individual. The arrow from *a* to *$* in the formula (Figure 5) makes clear that this object *a* plagues the subject, which again creates the move from *$* to S1. Thus, what is the capitalist discourse's answer to the discontent that previous consumption entailed? more consumption! As mentioned above, in Lacan's view the continuous circulation within the circuit of the capitalist discourse burns the subject out (Lacan, 1972, p. 48). What really gets consumed in capitalist discourse is desire itself: “the subject is suspended in the uncodable terrain of a contradictory circularity between success and failure, satisfaction and emptiness, limitless credit and limitless debt” (Mura, 2015, p. 170).

Just like the capitalist discourse, in the discourse of the hysteric the subject is also affected by *a* (Figure 6). Yet, contrary to what takes place in the discourse of the capitalist, *$* is also determined by S2. This means that in the discourse of the hysteric unconscious knowledge with a status of surplus-jouissance determines the subject. In the capitalist discourse, by contrast such determination remains absent. Indeed, under “capitalist exploitation” we are all “proletarians” because we are dispossessed from our knowledge, says Lacan (1969–1970, p. 33). Here, the knowledge that makes up the unconscious, and that determines the subject gets side-tracked: “What, in a type of subversion, gets returned to him is something different—master's knowledge” (Lacan, 1969–1970, p. 33). As Bruno (1993) indicates, this is why the agent in the capitalist discourse (*$*) is not so much the divided subject, with his unconscious from the discourse of the hysteric, but an individual who is plagued by a jouissance-related element (*a*), which is not determined by unconscious knowledge. Lacan qualifies this individual who gets dispossessed of his unconscious knowledge as a proletarian (Declercq, 2006).

The capitalist discourse implies a particular relation between the subject (*$*) and the object (*a*), which, as the arrows indicate, is the only element affecting the subject. Living in terms of the market of services and solutions (S1 → S2) does not gratify the subject, as was anticipated by the move from *$* to S1, but stirs corporeal tension. Consumption does not satisfy demand or desire, but exalts and exhausts the individual, who is haunted by an object that makes a strong appeal onto the subject.

An example of such a relation can be found in the gambler's relation to his game. The gambler knows that the game will not provide ultimate gratification, yet, at the same time he denies this lack and feels attracted to the glamor the game implies. Bjerg (2009, p. 59) describes this well: “he [the gambler] ascribes to the game a certain gaze by which the world of objects looks back at him. In the expression, ‘fortune smiles at him,’ there is the idea of a certain power (fortune) addressing (smiling at) precisely me. In this way, the game sees something special in the gambler, which is not otherwise visible.” The gambler fetishizes the game, which appeals to him and drives him to play the game. More broadly, the same holds true for capitalist consumption. The consumer knows rationally that consumption doesn't make him happy, yet at the same time the consumer feels attracted by something sublime, which he hopes to get hold of via the market (S1). Marketing and publicity exploit this fetishization. What the consumer meanwhile doesn't realize is that what makes him dream of the sublime is a lost cause (*a*) that cannot be recuperated by means of discourse.

From a clinical perspective, this shift from a master discourse to a capitalist discourse is evidenced in certain changes in contemporary psychopathology, and has brought about much discussion among Lacanians regarding, what they call, contemporary symptoms (e.g., Miller, 1993; Loose, 2002; Verhaeghe, 2004, 2014; Voruz and Wolf, 2007; Goldman-Baldwin et al., 2011; Redmond, 2013). Such symptoms, including addiction, panic or borderline states of functioning, are not thought of as metaphorical constructions that need deciphering, but as subjective expressions of, and reactions to, overwhelming surplus jouissance (i.e., mentally devastating corporeal tension). In other words, these symptoms no longer primarily reflect conflict and impossibility in relation to the other, but crises in response to a confrontation with the fundamental non-rapport.

## Lacan's solution: the saint

To what extent does psychoanalysis offer a way out of the capitalist discourse, and can it subvert the consumer's particular relation to the object *a*? In Television (1974), an edited version of a television interview with Jacques-Alain Miller, Lacan's reflection on the figure of “the saint” is most interesting at this respect (see also Bruno, 2010). In a section of this complex text, Lacan (1974, pp. 19–20) compares the psychoanalyst with the saint and concludes the following: “The more saints, the more laughter; that's my principle, to wit, the way out of capitalist discourse—which will not constitute progress, if it happens only for some” (Lacan, 1974, p. 20). This comment not only underlines the social inequality that capitalism implies, but stresses that contemporary subjectivity does not necessarily have to remain caught in the logic of the capitalist discourse. Indeed, in his view psychoanalysis offers the possibility of a way out of the maddening journey that the fifth discourse entails. On this point he makes the following remark: “So let's turn to the psychoanalyst and not beat about the bush …Because there is no better way of placing him objectively than in relation to what was in the past called: being a saint” (Lacan, 1974, p. 19). The saintly behaviors he has in mind have nothing to do with being compassionate or doing good. Lacan had already clearly differentiated such ethics of charity from the ethics of psychoanalysis during a discussion of the Christian Saint Martin, in Seminar VII (1959–1960). Later, in *Television*, Lacan returns to this idea: “A saint's business, to put it clearly, is not *caritas*” (Lacan, 1974, p. 19).

Echoing writings on the Taoist sage, as well as the seventeenth century philosopher Baltasar Gracián, Lacan situates the saint's actions relative to the idea of detachment: “the saint is the refuse of *jouissance*” (Lacan, 1974, p. 20; Dulsster, 2015). Thus, he situates the psychoanalyst's actions outside of the market of demand and supply, and outside the game of exaltation and disappointment. Psychoanalytic work should refrain from responding symmetrically to the solution-seeking consumer, and radically put aside the illusory mode of gratification implied in capitalist discourse.

The solution he puts forth consists of firmly holding on to the discourse of the psychoanalyst. This means that on the one hand the analyst should incarnate the object *a*, and personify the lost cause or abject-side of the object *a*: “he acts as trash” (Lacan, 1974, p. 19). Indeed, the analyst should not be stirred by the object, like the consumer is (*$*), and not present himself as the sublime solution (S1) for the crisis of the subject. Rather, the analyst takes into account the deadlock of the non-rapport, and challenges the subject to approach the object *a* in a different way. Hence Lacan's reference to the laughter of the saint: laughter bears witness to the evanescent nature of surplus-jouissance. The saint's laughter makes clear that properly dealing with the object *a* does not reside in the endless attempt to obtain a solution, but in finding a sinthome or savoir-faire in relation to its plaguing surplus-jouissance (see e.g., Verhaeghe and Declercq, 2002; Voruz and Wolf, 2007).

On the other hand, and closely connected to the object *a* position he occupies, Lacan suggests that the psychoanalyst should create a platform where the structural division of the subject can be articulated, which corresponds to the position of *$* at the place of the other in the discourse of the psychoanalyst. In capitalist discourse, subjective division is believed to be accidental and corrigible. Psychoanalysis, by contrast, assumes that subjective division is inherent to human existence and indicative of the unconscious. Therefore, the psychoanalyst occupies the position of the object: “So as to embody what the structure entails, namely allowing the subject, the subject of the unconscious, to take him as the cause of the subject's own desire” (Lacan, 1974, p. 19).

Both in the capitalist discourse and in the discourse of the psychoanalyst a direct relation between the object *a* and the divided subject can be observed (*a*→*$*). Yet, as Bruno (2010, pp. 207–208; 259–260) indicates, the respective positions differ, and the connecting arrow has a different status. In the discourse of the psychoanalyst a connection is made between agent and other (indicated by the horizontal arrow), along which a relation marked by non-rapport is installed. In this discourse *a* is manifested as a guarantee for the gap in *$*. In the capitalist discourse, by contrast, *a* is a haunting surplus that provokes *Unbehagen* in the subject and motivates the continuous purchase of new solutions.

## Capitalist discourse in clinical practice: a blessing or a curse?

How do these reflections translate into clinical work? I will explore this question with two clinical vignettes. The first, Nick, concerns a case that I situate in the clinical structure of obsessional neurosis. The second, Marc, concerns a case that bears witness to the clinical structure of autistic psychosis (Strubbe, 2011)^11^. In discussing these vignettes I will not discuss the details of the diagnosis. What the cases will hopefully illustrate is that, clinically speaking, capitalist discourse can both corrupt and/or protect the subject. At first sight this contradicts Lacan, who emphasized the corrupting impact of capitalism. Yet, in the case of psychosis, where an a priori exclusion from discourse stands to the fore, this might be different.

When discussing the capitalist discourse, Lacan presents it as a mode of relating to others that, at least partly, replaces the traditional discourse of the master. The capitalist discourse transforms the way in which the subject traditionally takes shape, and gives rise to new kinds of symptoms, as well as consumerist requests for help. What is characteristic of the consumerist request for help is that it ignores unconscious truth. In the discourse formula this is reflected in the position of the left arrow. Indeed, in neurosis, the main challenge in the clinical work consists in bringing the analysand to the point of exploring the unconscious determinants of subjective distress, thus implying a turn to the four discourses. After all, Lacan (1969–1970) indicates that the master discourse not only articulates the elementary social bond, but also expresses how the unconscious functions (Clemens and Grigg, 2006): signifiers (S1) that accidentally slip into speech challenge the knowledge (S2) one has about one's self, thus creating the dimension of a divided subject (*$*), as well as surplus-jouissance (*a*), prompting a continuous articulation of such signifiers (S1).

By contrast, in psychosis, the subject is principally positioned *outside* discourse. In his paper *l'étourdit* Lacan (1973b, p. 490) suggests that in psychosis no established discourse represents the subject^12^. This implies that in order to deal with the non-rapport, a semblance that provides stability has to be *invented* (Miller, 2004). Indeed, in psychosis a semblance that represents the subject, and enables a social bond is not installed discursively, implying that it needs to be actively established through an inventive act. By adopting a semblance, and adhering to it strictly, confrontations with the non-rapport are avoided. In my opinion, the capitalist discourse promotes a specific type of semblance that might function as a support in psychosis: the persona of the consumer who checks the market for solutions that might solve dissatisfaction. Capitalist discourse opens a market with solutions for distress, thus avoiding a confrontation with the fundamental non-rapport and with basic questions of existence, like “who am I” or “what do I want?” In case of psychosis such avoidance is functional since a signifier for addressing these issues is lacking, as Lacan's hypothesis of foreclosure makes clear (Lacan, 1959; Vanheule, 2011; Redmond, 2013). Conversely, when the semblance is challenged radically, and no longer functions as the hinge around which identity can turn, the subject is reduced to an object in relation to a voracious jouissance, which is unbearable.

In what follows I explore these ideas via two cases.

Nick is a successful 35-year-old architect, initially consulting me because of two symptoms he seeks relief from: he suffers from ADHD, which was first noticed by his ex-wife and then diagnosed by a psychologist, and he suffers with sexual problems in relation to his current partner, which, as he explains, might be a side effect of the medication (Ritalin) he takes. Concerning his life history he mentions a divorce about 1 year ago, indicating that he still maintains excellent relations with his former wife. Boredom made them split up he says. Medication helped him realize that he is often distracted, and suffers from difficulties organizing his life.

With respect to his ADHD, Nick particularly complains of an inability to concentrate for long times, which interferes with work and with social relationships. He is perpetually distracted and agitated, and it is for this agitation in particular that he wants to obtain a solution. Indeed, at first a problem-solving question stands to the fore: Nick wants to counter the corporeal tension that disturbs him. Nick is willing to engage in talking therapy, but ultimately he wants an answer: questions he frequently returns to include “do I really have ADHD?,” “is the medication good for me?,” “how can I handle the ADHD?” Thus considered Nick's attitude differs from the one Freud observed in *Studies on Hysteria*. Using hypnosis, Freud wanted to address specific symptoms that his patients suffered from, but instead of focussing their attention on the symptom, they talked elaborately about surrounding experiences, which eventually made Freud change his method. Nick's attitude is different in that, on the one hand, he is willing to talk, but on the other hand, he expects that after the talking I will offer him a solution, that is: an S1 that might solve the discontent (*$*) revolving around the disturbing excitation experienced (*a*). Within such logic subjective discomfort is something to be managed; not something that needs to be explored in detail because it could reveal who one is, hence the limit in the desire to talk.

What is crucial to analytic work in the context of neurosis is that we aim for a shift in the relation to the consumerist demand for solutions. By continuously inviting the analysand to speak about situations associated with the experience of discord, a shift toward the discourse of the hysteric is realized. Such a shift implies that discontent about the non-rapport in relation to the other is no longer buried under a maddening search for solutions, but gradually brought to the fore as a point around which the subject is positioned. Indeed, in the discourse of the analyst the analysand's discontent is taken seriously, with the aim of exploring key signifiers around which the analysand articulates choices in life.

As new issues come to the fore, conflict is induced in his self-experience. First Nick expresses a deep anxiety about failing in relation to other people, particularly his ex-wife, his current partner, and his mother. He wants things to go well, avoids conflict, and describes himself as “a pleaser.” He continually tries to second guess what other people want and then takes action, with the aim of giving people what he imagines they want. Nick almost literally aims at wanting what others want, and at fulfilling this demand, which, as discussed above, bears witness to how desire is shaped under capitalism. For example, when cooking in the evening he never knows what he wants and searches for clues concerning the dish he presumes the other wants. Similarly, he explains, that up until then, he never expressed preferences for holiday destinations. What he would do is listen to his wife's preferences, and then plan their vacations around this, telling himself that he wants what she wants. The semblance of subjective preference or discord (*$*) observed in his wife is not brought into a dialogue within the social bond, as is the case in the discourse of the hysteric. Rather, Nick identifies with it, and aims at immediately saturating it with an answer. Along that way he not only mortifies desire, by reducing it to the status of a demand, but also avoids expressing his own preferences, which would present him in a desiring position in relation to others. “I don't want to disrupt the other's pleasure” he says. Paradoxically, this search for gratification does not have a long-standing gratifying effect, but provokes a feeling of unease and a sense of boredom in close relationships.

As similar situations return in his speech, in subsequent sessions Nick realizes that in the years that they were married, he and his wife never argued. Moreover, during these years, his attention deficit symptoms, which his ex-wife observed, first came to the fore. When she was talking to him, he could not focus for a long time on what she was saying. His mind drifted to other topics, and he felt restless. In the end, he and his ex-wife lost intimate interest in each other. The mutual sexual attraction dissipated and without further dispute they broke up. Most remarkably what was absent from their relationship was an explicit struggle or search for ways of managing the non-rapport at the basis of their mutual connection. It can be argued that, in terms of Lacan's theory, the couple were organized along the lines of the capitalist discourse, with Nick in the role of the consumer who models his desire to the preferences of the other, and who looks for solutions that solve dissatisfaction. For him, dissatisfaction is not something to be discussed, but something to be managed. Yet, the solutions that he presents to his wife satisfy her desire only temporarily. Ultimately these solutions simply evoked further dissatisfaction on her side (S2), which confronted him with a surplus-jouissance (*a*) that he could not get rid of, as expressed in his restlessness and attention difficulties. However, the very act of addressing his habitual mode of relating to his wife implies a shift toward the discourse of the hysteric. His mode of relating to her is no longer self-evident to him, but becomes an object of inquiry and reflection.

In terms of his sexual problems in his current relationship, which Nick gradually begins to discuss in detail, a similar logic can be discerned. On the one hand, Nick expresses feelings of happiness with his new partner, indicating that meeting her has truly enriched his life, and stimulated his creativity. On the other hand, he suffers from falling short sexually. While discussing the situation in detail, it becomes clear that actually his new partner has strong sexual yearnings, and often longs for sex while he is not in the mood for it. Typically, Nick succumbs and starts cuddling her, which bears witness to his attempt to mold his desire to hers, and to engage in a search for gratification. Yet, at this point his body fails to obey his own will, resulting in erection difficulties. These difficulties bear witness to his own unconscious desire, which is not satisfied by the act of intercourse. The erection difficulties lay bare the extreme satisfaction-oriented position he occupies. The physical inability prevents him from occupying his habitual position on the market of human demand and supply, which provokes a state of crisis (*$*), for which he considers taking medication to treat erectile dysfunction.

Nick's initial position in the transference resembles a capitalist discourse, asking me to provide a solution for his symptoms. Yet, via the psychoanalytic sessions, a change in position is evident. A number of interventions, like “Why concentrate on being a pleaser?” or “you will never get rid of your symptoms unless you learn how to have conflict,” surprise him. On the one hand the analyst is not in the problem-solving position that he anticipated him to be: symptoms are not tackled by means of precise solutions. On the other hand he suddenly recognizes the satisfaction-oriented position that he usually ends up in, and he starts to wonder what it is that determines his actions. At this point his speech resembles the discourse of the hysteric: Nick stops taking the ADHD medication; and starts talking about characteristic relations and incidents in the family, about the turbulent period he goes through with his partner, and about his job as an architect. Holding on to analytic discourse myself, Nick observes and starts to explore the details of his tendency to please, gradually shifting to other discourses in relation to others.

Nick's functioning along the lines of the capitalist discourse bears witness to denying subjective division, which is countered by a strict use of analytic discourse. This enables a switch to the hysteric discourse, in which Nick begins to question his own mode of subjective functioning.

In Marc's case, by contrast, the use capitalist discourse has a stabilizing effect.

Marc is a 43-year-old ICT specialist, with a competitive job in an international company. One of his hobbies, which he qualifies as an obsession, is watching Japanese animation, also called anime. However, recently, an event turned his life upside down: the police searched his house and found pornographic images of minors on his computer. Marc is shocked and overwhelmed by panic. He didn't realize that he was doing illegal things. Often Japanese animation films have a sexual component, and by downloading these films, pornographic images that circulate under the alias “anime” were also copied to his PC. Marc is aware of this problem, but removed these pictures. Yet, the police traced his activities and put him under surveillance. Marc is overwhelmed and confused. After all, the idea of living within the boundaries of the law is a fundamental principle he strongly holds on to. The subjective crisis Marc lives through makes clear that anime functioned as a master signifier, helping him to position himself in the world.

Professionally, Marc is the head of a department within an ICT company. In running his team Marc starts from the idea that just like algorithms make a computer run, social relations are made up of algorithms. All one needs to do is apply the correct social algorithm in the correct context. Along that way he learned how to make eye contact; observed that in discussing things one shouldn't insist too long; and found out that humor means saying the opposite of what you intend. The algorithm is a master signifier (S1) that he, qua consumer (*$*), easily adopts because it is presumed to reveal the truth about how human relations function. However, his algorithmic approach is not perfect. One day a colleague asked him how he feels when working under strong pressure. Marc never thought about that issue, and was baffled by the question.

Marc states that visual images are important to him, and that he has a photographic memory. One day he had to make an apparatus consisting of more than 30 pieces. He had no manual, and at first he couldn't do it. Suddenly when driving his car he saw the solution, and could fix the machine in 30 s. “Being autistic has its advantages” he says. Importantly, at this level anime had an important function for Marc. He explains that visual stimuli are often overwhelming, and that he needs anime images to process other images he is confronted with. For example, social relations often confuse him, and anime helped him to organize his world: “it was my model of the world. Anime often deals with the topic of love. I learned how to relate to a woman, like by giving her a flower, and things like that. Without animation I would never have met my wife.” The fictional universe of anime functioned as an instrumental framework that helped him manage relationships with other people. Considered from the perspective of discourse theory, anime was an S1 that reflected the truth of how human relations are organized, and provided him with instrumental knowledge (S2), that helped him to identify with the role of the consumer (*$*) and thus avoid brutal confrontations with the other.

As he didn't have anime at his disposal anymore, Marc ended up in chaos. Suddenly, actions of the other provoke a highly uncomfortable and haunting tension. Surplus-jouissance (*a*) urges him to find new ways of behaving as a consumer (*$*) on the market of supply and demand. During our sessions, he aimed at “speaking about emotions in a non-emotional way.” Indeed, Marc has a precise idea about our sessions: they should be a sounding board for rational reflection on emotional issues. The function he attributes to the analyst consists of offering a platform where rational solutions can be found, far removed from the subject supposed to know which motivates transference in neurosis. His approach fits the logic of Lacan's capitalist discourse: sessions with the psychoanalyst make up a marketplace where he looks for suitable alternatives for the lost Japanese animation.

Most particularly, Marc is troubled by the fact of living with his wife. He says that her “orders” are often contradictory, which is extremely difficult since he cannot but try to fulfill all of her demands. In terms of Lacan's capitalist discourse, Marc aims at finding an S1 for all of his wife's demands, with which he as a subject identifies (*$*). Yet, since losing the framework of anime, his solutions (S1) are also lost, which leaves him with no escape form the surplus-jouissance (*a*) that overwhelms him: “her orders literally consume me” he says. Indeed, without the framework of anime he becomes identified as the object of jouissance for the other, which is an unbearable position that can only be solved by inventing a new solution.

The analyst's answer to Marc's problems and demands doesn't consist of installing a hysterical discourse. Given the autistic structure in Marc's functioning, questioning his subjectivity is not seen as a way out of the chaos that he is confronted with. Instead, the analyst focuses on finding a new instrumental framework such that Marc can again deal with the non-rapport that he is confronted with in relation to his wife. During the sessions Marc evaluates different solutions. He proves to be inventive, but clings to the analyst's opinion in terms of validating the adequacy of what he invents.

Stand-up comedians provide a first alternative to anime. He observes that comedians often joke about the difference between men and women, and learns from them that women often ask for the things a man cannot give. This idea has a pacifying effect, helping Marc to deal with his wife's demands. Next to that he holds on to the idea that women's menstrual cycle explains how they feel and behave. This insight helps him to frame his spouse's emotional expressions. Finally, Marc begins to classify his wife's demands according to the system they use at his place of work. When clients present demands and requests they are classified hierarchically in terms of their urgency. “Major” demands require direct action, while “trivial” demands can be solved when no urgent requests are waiting. To his surprise Marc concludes that not all “orders” his wife articulates are equally urgent. Depending on formal characteristics, like the pitch, tempo and timing of what she says he is able to interpret the urgency of what she articulates. Actions that were capricious at first now become predictable, which solves the experience of chaos that he was confronted with. In line with the logic of Lacan's capitalist discourse the classification system helps Marc to frame his wife's capricious “orders” as demands, with which he identifies (*$*). Now he activates instrumental solutions (S1) that provide him with knowledge (S2) concerning the other. By enveloping the other with an S2, the other's jouissance which reduces him to a mere object is avoided.

## Conclusion

In this paper I discussed Lacan's (1972) reflections on the capitalist discourse, a fifth discourse introduced after the classic four discourses. In his view, the capitalist discourse partly replaced the discourse of the master, which implies an important shift in how the subject and the other deal with their fundamental non-rapport. The capitalist discourse implies identification with the semblance of the consumer who shops for solutions that make up the truth of his discontent. However, artifacts offered on the market cannot provide the hoped for satisfaction and instead produce surplus-jouissance, which provokes *Unbehagen* or crisis at the level of the individual. In neurosis the discourse of the capitalist functions as an attempt to ignore the sexual non-rapport and the dimension of the unconscious. Consequently, psychoanalytic work with patients caught up in this discourse implies a shift to the discourse of the hysteric, and thus how the individual relates to symptoms and complaints. Psychosis, by contrast, is marked by an a priori exclusion from discourse. In psychosis, the capitalist discourse might provide a way out of the tricky position of being a mere object of jouissance to the other. Indeed, the right-hand downward arrow in the capitalist discourse could be interpreted as representing the position of being an object *a* in relation to a veracious jouissance. Consumerist ways of relating to the other might offer the individual a semblance and thus a way out of this deadlock, such that a mode of relating to others can be invented.

This line of reasoning implies that the capitalist discourse should not be disqualified *per se*. Lacan's discussion of the capitalist discourse, by contrast, is inherently critical. However, as he qualifies the discourse of the capitalist as a contemporary mutant of the discourse of the master, it seems that Lacan only focused on neurosis, and did not discuss the implications for psychosis. I believe that if we take into account both neurosis and psychosis, Lacan's capitalist discourse offers a promising framework for understanding clinical problems in which the so-called “administration of jouissance” stands to the fore (Loose, 2002). As the cases discussed in this paper illustrate, problems such as ADHD and autism obey this logic, but addictions, eating disorders and some anxiety disorders might be fruitfully studied from this perspective.

## Author contributions

The author confirms being the sole contributor of this work and approved it for publication.

### Conflict of interest statement

The author declares that the research was conducted in the absence of any commercial or financial relationships that could be construed as a potential conflict of interest.

## Footnotes

^1^For example, this idea is addressed in the R-schema (Lacan, 1959) and in the discourse of the master from Lacan's discourse theory (Lacan, 1969–1970).

^2^Other discussions of Lacan's discourse theory can be found in Clemens and Grigg (2006), Bracher (1994), Verhaeghe (1995), and Žižek (2006).

^3^As Bianchi (2012, p. 116) indicates: “Discourse is in fact etymologically derived from the Latin *discursus:* i.e. to run from a place to another. The Lacanian discourse is in fact a structural system of places and relations that regulates the interaction between these terms, i.e. their *currere* from a place to another and their mutual exchange of positions.”

^4^At first Lacan denotes the position of the ‘agent’ as the position of desire (Lacan, 1969–1970, p. 93).

^5^In Seminar XVI Lacan describes the object *a* as follows “I have said that the *a* is that which conditions the distinction between the *I* qua support of the field of the Other, which can add up as the field of knowledge, and the “*I* of jouissance” (1968-69, p. 135, my translation). In this context, the “*I* of jouissance” refers to the fact that as a corporeally living being I'm marked by the drive. “*I* qua support of the field of the Other,” in its turn, refers to the fact that I make use of language to signify myself and the world.

^6^A different interpretation of hypermodern discourse, which does not follow Lacan's fifth discourse, but takes Lacan's discourse theory as its point of departure, has been provided by Jacques-Alain Miller (2005). Bryant (2008) in his turn situates the capitalist discourse as just one of four discourses in the universe of capitalism, the others being the discourse of bio-power, the discourse of immaterial production, and the discourse of critical theory.

^7^Not all authors who discuss Lacan's fifth discourse take into account these three mutations. For example, Bryant (2008) only takes into account Lacan's suggestion that in capitalist discourse *$* and S1 exchange places, but not the two other mutations he points to. Such a partial reading has important weaknesses. For example, the decline of the social bond and the changing position of truth, which are proper to the regime of capitalism (see e.g., Miller, 1993; Žižek, 1999) remain underexposed.

^8^In French the text goes as follows: “une toute petite inversion simplement entre le S1 et le *$*, qui est le sujet, ça suffit à ce que ça marche comme sur des roulettes, ça ne peut pas marcher mieux, mais justement ça marche trop vite, ça se consomme, ça se consomme si bien que ça se consume.” In my translation the reference to consumption is lost, yet translating the French verbs “se consommer” and “se consumer” as “to consume” largely misses the clue of the sentence, which is the idea that the very success of this discourse is also its own fiasco.

^9^Lacan (1969–1970, p. 92) closely links the idea of the market to the master signifier.

^10^Lacan (1969–1970, p. 110) indicates that the imperative of science comes down to “Keep on knowing in a certain field.”

^11^In the original case study the link with Lacan's discourse theory in general, or with capitalist discourse in particular is not made.

^12^“l'hors-discours de la psychose” (Lacan, 1973b, p. 490).
